# Genetic and genomic analyses of tree architectural traits in *Hevea brasiliensis* revealed genes underlying QTLs linked to key developmental processes

**DOI:** 10.1371/journal.pone.0344014

**Published:** 2026-03-17

**Authors:** Nur Eko Prasetyo, David Lopez, Fetrina Oktavia, Eka Tarwaca Susila Putra, Pascal Montoro

**Affiliations:** 1 Indonesian Rubber Research Institute, Unit Bogor Getas, Java, Indonesia; 2 CIRAD, UMR AGAP Institut, Montpellier, France; 3 UMR AGAP Institut, Univ Montpellier, CIRAD, INRAE, Institut Agro, Montpellier, France; 4 Indonesian Rubber Research Institute, Banyuasin, South Sumatra, Indonesia; 5 Faculty of Agriculture, Universitas Gadjah Mada, Yogyakarta, Indonesia; Dev Bhoomi Uttarakhand University, INDIA

## Abstract

The architectural characteristics of rubber trees are increasingly important in the context of climate change. Branching, canopy shape and growth pattern have to be adapted to monocropping and intercropping systems to foster latex and wood production, wind-tolerance, light availability and microclimate for intercrops, and soil stability and fertility. This study aimed to identify key architectural traits that could be used in breeding programs as well as chromosomal regions underlying QTLs that could be targeted for marker-assisted selection. Five quantitative (height of bole, trunk girth, estimated bole volume, number of terminal branches and apical shoots) and five qualitative (tree straightness, axillary shoot score, type of crown and axillary shoot, diameter of axillary shoot) architectural traits were phenotyped in a segregating population derived from clones PB 260 and SP 217. The frequencies of categories for each qualitative architectural variable were analysed as quantitative variables. A principal component analysis performed with these traits showed that trunk girth, estimated bole volume, round crown and medium diameter of axillary branches are negatively correlated with small diameter of axillary branches and conical type of crown. Seven architectural variables have heritability greater than 0.50. Twenty quantitative trait loci and their underlying genes and functions were pinpointed using the high-density genetic map previously constructed and an improved high-quality genome of the parent clone PB 260. Of the 680 genes found in chromosomal regions under QTLs, 19 genes have a function directly involved in plant development such as transcription factors related to the regulation of shoot apical meristem (SAM) and vascular cambium activity (WUSCHEL). A literature review was also conducted to provide additional insights into tree architecture and its impacts on agricultural systems. This first genetic analysis of architectural traits in rubber revealed that seven traits (trunk girth, bole height, estimated bole volume, number of apical branches, diameter of axillary branches, number of terminal branches and type of crown) could play a major role in rubber breeding both for monoculture and agroforestry single and double row systems. Chromosomal regions harbouring developmental genes could be used to develop specific strategy of marker-assisted selection.

## Introduction

Tree architecture significantly influences agricultural productivity and ecosystem resilience either in monocropping or intercropping systems, especially in the context of climate change [1,2]. Tree structure – comprising branch arrangement, canopy shape, overall growth pattern and phyllotaxy – affects how they interact with their environment. It offers critical benefits such as optimising light availability, which is essential for photosynthesis and plant growth of associated crops [3], providing shade [4,5], optimising microclimate conditions by maintaining ideal temperatures of soil and air, enhancing soil fertility indicators [5,6], and enhancing biomass production [7]. In addition, some specific tree architectures can minimise wind damage in both monoculture and polyculture systems [8,9]. Tree architecture is deeply influenced by both primary and secondary growth mechanisms [10,11]. Genetic [12–14], and functional studies [15–17] were mainly carried out in temperate fruit trees and forest trees such as poplar, eucalyptus and conifers to better understand fruit and wood production and little is known about tropical trees.

*Hevea brasiliensis* remains an essential source of natural rubber that is crucial to numerous industries [18]. Natural rubber possesses outstanding characteristics and significant potential for further enhancement [19]. Native to rainforests in the Amazon region, rubber trees grow in tropical and subtropical areas. Rubber plantations are mainly monospecific, but rubber-based agroforestry remains well developed on smallholdings, particularly during the immature period. Climate change poses significant challenges to rubber cultivation, impacting growth, productivity and the long-term viability of *Hevea brasiliensis* [20]. Rising temperatures, altered rainfall patterns and increased frequency of extreme weather events threaten the stability of rubber yields. To address these challenges, researchers and growers are exploring the adaptation of rubber cropping systems and clones to improve farmers’ resilience and the sustainability of rubber production [21]. One key approach is the development and deployment of climate-resilient rubber clones [22]. In addition to latex yield, wood production and leaf disease resistance, breeding programmes take traits for tolerance to abiotic stresses such as drought [23], water-use efficiency [24] and new diseases [25] that may be exacerbated under changing climatic conditions into account. Another strategy is the diversification of cropping systems, particularly through rubber-based agroforestry systems [26]. These systems aim to balance production efficiency with ecological sustainability, offering a promising approach to mitigate the environmental impact of rubber cultivation while maintaining its economic importance [27]. Crop association under trees can mitigate the impacts of climate change by reducing wind velocity [28], maintaining internal microclimate stability [29], mitigating soil erosion [30,31], redistributing rainfall [32], enhancing carbon sequestration [33], restoring degraded land, promoting ecosystem stability [34] and improving the resilience of agricultural systems [2]. Moreover, this system can facilitate agroforestry diversification and accelerate investment returns [35,36].

The architecture of *Hevea brasiliensis* follows Rauh’s model, characterised by a monopodial trunk with rhythmic growth patterns that produce branches in verticillate or sub-verticillate arrangements [37]. The upright (orthotropic) branches resemble the trunk, and flowering occurs laterally along both branches and the trunk. Although orthotropic branching is mainly described in rubber trees, plagiotropic branching is observed with branches growing parallel to the ground. Juvenile trees exhibit a single vertical stem with long internodes, transitioning to branching as they mature, forming a candelabra-like shape with clusters of branches. The rhythmic growth of the tree is marked by distinct periods of active growth and rest, leading to the formation of identifiable growth units. Lower branches often die off in dense plantations due to shading, while those in open areas may develop larger, horizontal branches. The trunk typically grows 3–6 meters in height before branching, but may branch earlier (at 1.5 to 1.8 meters) if damaged. Rubber trees are often cloned by grafting. These high-yielding clones often have thinner, cylindrical trunks with less base thickening than seedling trees, probably due to stock variation or branching habits. These unique traits of *Hevea* reflect its adaptability and response to environmental conditions [38].

The rubber tree architecture is a key factor in its adaptation to climate change and cultivation systems in particular to control wind damage, microclimate under trees and light availability for associated crops in the case of agroforestry. Rubber planters prevent wind damage by using wind breakers, high-density planting [39–41] and pruning to control tree architecture [42]. Attempts to breed wind-tolerant rubber clones have been made to cope with the cost of tree maintenance by pruning. In China, particularly in Hainan and Guangdong provinces where typhoons present a major challenge to rubber cultivation, wind resistance has been a key focus for rubber clone improvement since the 1950s, leading to the development of resilient clones such as Haiken 1, Wenchang 217, Wenchang 11, Reyan 7-33-97, Haiken 2, Wenchang 33−24 and Xuyan 141−2, which have proven their durability over decades of planting [43]. Although phenotypic diversity for architectural traits has been observed, the incorporation of tree architecture into breeding objectives remains underexplored. The main architectural traits observed by breeders are the compactness of the crown [7] and short branches with narrow angles [9,44] that can therefore reduce wind exposure to minimise wind damage.

Given genetic studies related to architectural traits in rubber have mainly focused on trunk girth and height [45–53], this study aimed to phenotype a F1 population for a large combination of architectural traits in order to identify key traits that could be used in breeding programs for different cropping systems (monoculture and agroforestry) in a context of climate change. The phenotyping was conducted on a segregating population derived from clones PB 260 and SP 217 [54]. Five quantitative (height of bole, trunk girth, estimated bole volume, number of terminal branches and apical shoots) and five qualitative (tree straightness, axillary shoot score, type of crown and axillary shoot, diameter of axillary shoot) traits were phenotyped. The frequencies of categories for each qualitative architectural variable were calculated and used as quantitative variables. All of the quantitative data were used for principal component and genetic analyses. Chromosomal regions underlying quantitative trait loci (QTL) were identified in order to provide information for developing a marker-assisted selection strategy. Using the high-density genetic map and high-quality genome of the parent clone PB 260 [54], 20 QTLs were identified for four quantitative and three qualitative traits. Nine chromosomal regions harbouring genes underlying these QTLs showed functions associated with plant development. By combining these findings with insights from a literature review, the results offer significant potential for advanced breeding programmes. These traits were discussed with regard to intercropping and wind tolerance. These insights pave the way for creating superior rubber clones, adapted to economic and ecological needs.

## Materials and methods

### Plant material

A small-scale clone trial consisting of 202 genotypes resulting from the crossbreeding of PB 260 and SP 217 was established at the Sembawa Research Centre in South Sumatra [54]. Clone PB 260 was used as female clone as trees of this clone produce more flowers available for hand-pollination than those from clone SP 217. This trial was designed as a randomised complete block design. The planting density was 550 trees per hectare (6 m x 3 m). These genotypes were planted in November 2016 in five different blocks, with each block consisting of two trees per genotype. All the genotypes in the blocks were surrounded by border trees to avoid any border effects.

### Observation and data collection

The architectural traits were observed on seven-year-old trees, as their architecture is well established and competition between trees is not yet sufficient to affect the canopy. Of the 202 genotypes in the F1 population planted in the small-scale clone trial (SSCT), 189 were characterised in this study, with the remainder lost due to the death of some trees. Architectural traits were observed on 1,855 trees in this trial from September to November 2023 corresponding to living trees of the 191 accessions (genotypes of the F1 population and parent clones).

No pruning was applied in this trial. All of the trees therefore maintained their original architecture in plantation conditions. Phenotyping was conducted by observing the characteristics of plant architecture. The observed variables were defined after a literature review (Table 1).

**Table 1:** Comparison of architectural terms for the canopy description used in this study and in the literature.

This study	Clement-Demange (1996)	Cilas (2004)	Gireesh & Mydin (2014)	Yun (2019)
Round (R)	–	–	globose	–
Oval (O)	–	–	–	–
Broom-type (B)	fan-shaped, brush-shaped	paintbrush	–	vase-shaped
Columnar (CL)	–	–	–	–
Conical (CO)	cone-shaped	outspread	–	–

Consequently, the F1 population was observed for five quantitative variables. (1) Height of bole (BH) is a variable that describes the height of the trunk sequence below the first branching terminal, measured from the point of grafting union to the first branching terminal. The unit of measurement was in meters (m) with two decimals. (2) Trunk girth (GIRTH) was determined by measuring the girth of the trunk at a height of 150 cm from the grafting union. The unit of measurement of this variable was cm with one decimal. (3) The number of terminal branches (BRCHTRM) was determined by counting the existing terminal branches in the apical shoots. (4) The number of apical shoots (APNUM) of each tree was observed, this number usually varies from one to four. (5) Estimated bole volume (EBV) was determined using the following formula:


EBV=BH×circle area of the BOLE

$$\;=\text{BH}\times \pi r^{2}$$

$$\;=\text{BH}\times \pi \times \frac{GIRTH}{2\pi }\times \frac{GIRTH}{2\pi }$$

Five qualitative variables were phenotyped. (1) The stand of the tree straightness (STRAIGH) had four categories, i.e., straight (STRAIGH_S), curved (STRAIGH_C), leaning (STRAIGH_L) and leaning and curved (STRAIGH_LC). (2) Axillary shoot score (AXSCO) was based on the number of axillary shoots per plant. There were three categories for this variable, i.e., abundant (AXSCO_A) when it consisted of more than nine terminal branches, medium (AXSCO_M) when it consisted of six to nine terminal branches, and little (AXSCO_L) when it consisted of less than six terminal branches. (3) Type of crown (TYCRO) was determined on the basis of the canopy shape, whether it was round (TYCRO_R), oval (TYCRO_O), broom-type (TYCRO_B), columnar (TYCRO_CL) or conical (TYCRO_CO) (see Table 1). (4) Type of axillary shoot (AXTYP) was determined by the growth pattern of axillary shoots, whether it was mostly orthotropic (AXTYP_MO), fairly between orthotropic and plagiotropic (AXTYP_F), or mostly plagiotropic (AXTYP_MP). (5) The diameter of axillary shoot (AXDIAM) was estimated visually after a period of caliper measurements to optimize the estimation. Trees were categorised based on the diameter of their branches into three distinct categories. Trees were classified as having small branches (AXDIAM_S) if all their branches had diameters of less than 5 cm. Trees were classified as having medium branches (AXDIAM_M) if they had at least one branch with a diameter between 5 and 10 cm. Lastly, trees were classified as having large branches (AXDIAM_B) if they had at least one branch with a diameter greater than 10 cm. This classification helped to assess and compare the structural attributes of different trees based on their branch sizes.

### Data analysis of phenotypic data

The LS means of quantitative variables BH, GIRTH, EBV, APNUM and BRCHTRM were obtained from the ANOVA using the average of data from the two trees per block. The frequency of each category of qualitative variables STRAIGH, AXSCO, TYCRO, AXDIAM and AXTYP was calculated from the ten trees per genotype or parent clone in the trial. These data stored in the S1 Table were used to perform a principal component analysis (PCA).

### Genetic analyses

Heritability was calculated using data from the five replicates of the randomised complete block design of the trial to estimate the variation due to environment. For quantitative traits BH, GIRTH, APNUM and BRCHTRM, the broad-sense heritability (H^2^) was calculated using R Studio (version 4.4.3) with the lme4 package for the mixed model equation [55]. The broad-sense heritability at the genotype level is expressed as:


$$\frac{Vg}{\left(Vg+\frac{Ve}{n}\right)}$$

where H^2^ represents the heritability at the genotype level, Vg is the genetic variance, Ve is the environmental variance, and n is the number of individuals per genotype.

For qualitative traits with several categories, H^2^ was calculated using a generalized linear mixed model (GLMM) using the R package glmmTMB, which supports multinomial models, and a data frame consisting of the 189 genotypes, the 10 individuals per genotype, and the categories of traits STRAIGH, TYCRO, AXSCO, AXTYP and AXDIAM. H^2^ was calculated as the proportion of total phenotypic variance explained by genetic variance based on the equation:


$${\text{H}}^{\text{2}}={\sigma_{\text{g}}}^{\text{2}}/{\sigma_{\text{g}}}^{\text{2}}+{\sigma_{\text{e}}}^{\text{2}}$$

where H^2^ represents the heritability at the genotype level, σ_g_^2^ the genotype variance and σ_e_^2^ the residual variance.

QTLs were detected using MapQTL6 (Kyazma B.V. 1996–2011, Wageningen, Netherlands). For quantitative traits, Best Linear Unbiased Prediction (BLUP) was calculated from the values of five replicates per genotype, while for qualitative traits the frequency of each category was directly used. The linear model applied was:


$${\text{Y}}_{\text{ijk}}=\mu +{\text{G}}_{\text{i}}+{\text{b}}_{\text{j}}+{\text{D}}_{\text{ij}}+\epsilon_{\text{ijk},}$$

where μ is the constant term, G represents the genetic effect, b denotes the block effect, D accounts for the genotype by block interaction, and ε is the residual error.

Three data files were used in the QTL analysis. The file.qua was generated in this study using the BLUP data. The locus genotype file (file.loc) and the SNP position mapping (file.map) were created by Ismawanto and collaborators, and were directly used for this analysis [54]. Since most of the categorical data of the F1 population did not follow a normal distribution, a non-parametric Kruskal-Wallis (KW) test was conducted. The Kruskal-Wallis (KW) statistical test is significant below the threshold of 0.01. Since many long regions were significant in this study, this threshold was increased to a K value greater than 10 and a p-value less than 0.0005 for the identification of QTLs.

### Analysis of chromosomal regions and underlying genes

In order to identify putative genes located in the QTLs, the genome assembly of clone PB 260 was improved by ordering contigs into 18 linkage groups with the help of the GBS linkage map [54] and LepAnchor [56]. The 18 linkage groups represent 96.3% of the total assembled contigs in terms of size. The remaining unplaced contigs were put together on a 19th contig separated by stretches of Ns. Sequence and annotation are available at https://zenodo.org/records/14218078. The identification of transposable elements was carried out with EDTA [57] and Inpactor2 [58]. Libraries were merged (without manual curation) and used for annotation using RepeatMasker (https://www.repeatmasker.org/RepeatMasker/) with default parameters. The total repetitive content represents 1,268,790,504 bp or 78.49% of the total assembly placed in lowercase nucleotides. *De novo* gene annotation was carried out using Helixer v0.3.2 cuda [59] with the “land_plant” lineage model run on Nvidia A100 GPU hosted at the MESO@LR platform. The resulting 40,263 predicted genes were searched for predicted functional annotation using InterProScan-5.67–99.0 [60]. Functional annotation was predicted for 36,613 genes, and 22,047 genes had associated Gene Ontology (GO) terms that served as background for enrichment tests. Genes located within QTL coordinates were extracted using Bedtools [61]. GO term enrichment was done by testing frequency discrepancy between GO associated with QTL genes vs. the background. Only GO terms with a family-wise error rate below 0.05 (FWER, i.e., adjusted p-value) obtained with the hypergeometric test function in the GOfuncR v1.22.2 package [62] were considered significant and reported with the associated genes. Genes associated with the so-called enriched GO terms are reported as ‘Genes with significant GO term enrichment’ in the results thus the number of significant GO terms enriched, and their associated genes are likely different.

## Results

### Data distribution of architecture variables

**Fig 1:**
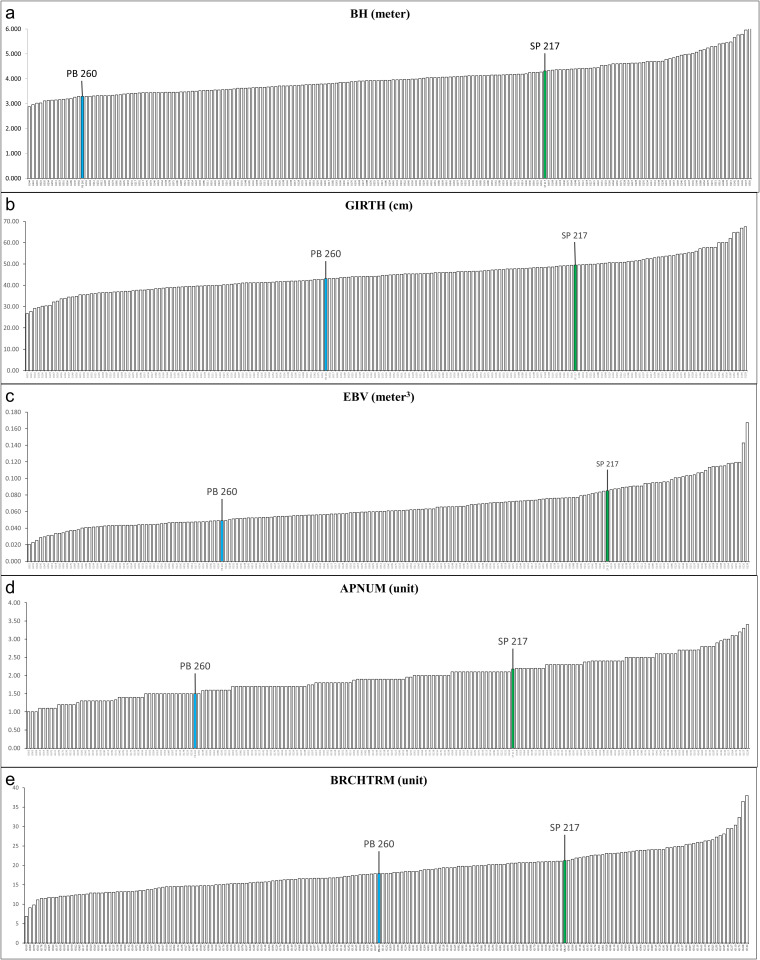
Quantitative values of some architecture variables for the parent clones, PB 260 (blue bar) and SP 217 (green bar), and the F1 population: (a) height of bole: BH; (b) girth of stem: GIRTH; (c) estimated bole volume: EBV; (d) number of apical shoots: APNUM; (e) number of branch terminals: BRCHTRM.

Data distribution of the F1 population consists of five quantitative architecture variables and five qualitative categorical variables. The quantitative variable data (i.e., BH, GIRTH, EBV, BRCHTRM and APNUM) is shown in histograms (Fig 1). BH values are 3.29 m for parent clone PB 260 and 4.31 m for parent clone SP 217, while for the F1 population the values range from 2.88 m (G144) to 6.25 m (G030). For GIRTH, the parent values are 43.08 cm for PB 260 and 49.47 cm for SP 217, while for the F1 population, GIRTH values range from 25.68 cm (G019) to 67.46 cm (G228). For EBV, the parent values are 0.05 m³ for PB 260 and 0.09 m³ for SP 217. For the F1 population, EBV values range from 0.020 m³ (G021) to 0.167 m³ (G228). APNUM values for the parent clones are 1.50 for PB 260 and 2.18 for SP 217, while for the F1 population, APNUM values range from 1.0 (G005, G065 and G099) to 3.4 (G228). For BRCHTRM, the parent values are 17.90 units for PB 260 and 21.25 units for SP 217, while for the F1 population, the values range from 6.9 unit (G019) to 38 unit (G144).

**Fig 2:**
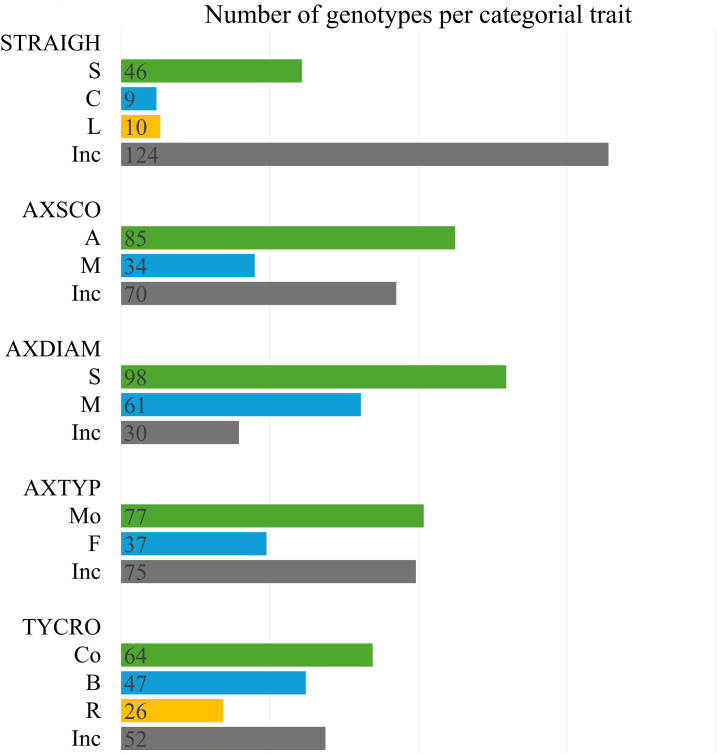
Distribution of qualitative architectural variables for the F1 population: (a) type of straightness: STRAIGH; (b) type of axillary branch diameter; (c) score of axillary branches: AXSCO; (d) type of axillary branches: AXTYP; and (e) type of crown: TYCRO.

The categories of qualitative architecture variables (i.e., TYCRO, AXTYP, AXDIAM, STRAIGH and AXSCO) were observed for the two parental clones and the F1 population (Fig 2). The ten trees of the same genotype can be assigned to different categories of a qualitative trait. The criterion for assigning a category by genotype is a consensus threshold of 60%. If several categories had a frequency greater than 25%, they were taken into account for the classification of the genotype. Otherwise, the genotype was classified as inconsistent.

The STRAIGH variable showed a large number of genotypes with inconsistent phenotype (124 genotypes) then 46 genotypes for S category, 9 for C and 10 for L. The AXSCO showed two distinct types, 85 genotypes for A category and 34 for M but genotypes with inconsistent phenotype were still important (70 genotypes). The AXDIAM variable also revealed two distinct types: 98 genotypes for S category and 61 genotypes for M. The number of genotypes with inconsistent phenotype is lower (30 genotypes). The AXTYP variable showed two main types: 77 genotypes for Mo category and 37 genotypes for F. This qualitative trait showed a large number of genotypes with inconsistent phenotype. The TYCRO variable showed three distinct types: 64 genotypes for the Co category, 47 for the B and 26 for the R. The number of genotypes with inconsistent phenotype was 52.

Overall, the distribution of qualitative architecture variables shows that inconsistent category ranges from 15.87% for AXDIAM to 65.61% for STRAIGH. AXSCO, AXTYP and TYCRO have 37.04%, 39.68% and 27.51% of genotypes respectively with an inconsistent phenotype. This high inconsistency suggests phenotypic plasticity, probably influenced by environmental factors or gene-environment interactions.

### Principal component analysis of architectural variables

**Fig 3:**
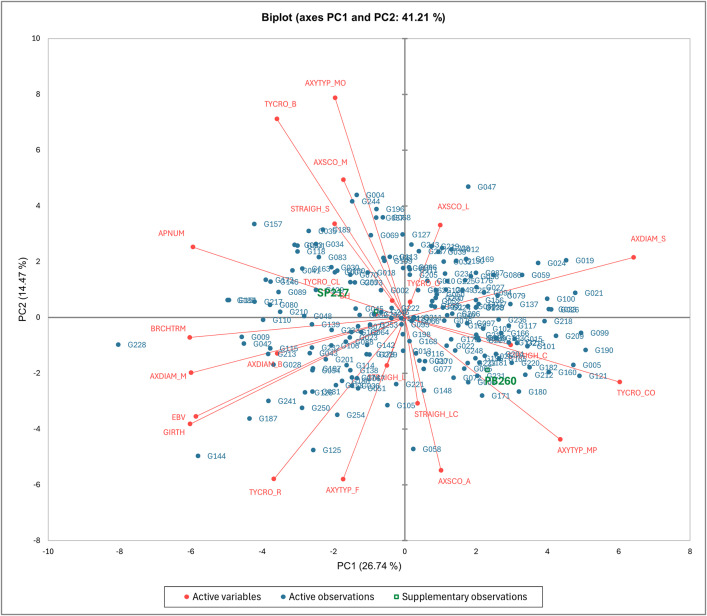
Principal Component Analysis (PCA) biplot of the ten architectural variables of the F1 population and parent clones.

The PCA was carried out with data from the quantitative variables and the frequencies of all the categories from the qualitative variables. The two principal components PC1 and PC2 had a total explained variance of 41.21% (Fig 3). PC1 explains 26.74% of the variance with two groups of variables negatively associated. TYCRO_CO and AXDIAM_S had positive factor loadings (respectively, 0.812 and 0.865), while BRCHTRM, GIRTH, AXDIAM_M, APNUM, and EBV had negative factor loadings (respectively −0.811, −0.810, −0.806, −0.799, and −0.788). PC2 explained 14.47% of the variance with two associated groups. AXTYP_MO (0.779) and TYCRO_B (0.705) were negatively correlated to AXTYP_F (−0.574), AXSCO_A (−0.543) and TYCRO_R (−0.574). The dispersion of genotypes on the PCA graph suggests an absence of major genetic structuring along the principal axes considered. Nevertheless, the correlation between certain phenotypic traits and these axes indicates that genotypes close to the vectors of these traits could be associated with their variability.

### Broad-sense heritability of the architectural variables and categories of the F1 population

The value of broad-sense heritability (H²) was calculated for each variable (Table 2). Seven variables with H² values greater than or equal to 0.50 are considered to have high heritability, i.e.,: AXDIAM (0.94), EBV (0.84), GIRTH (0.80), APNUM (0.60), BH (0.58), TYCRO (0.53), and BRCHTRM (0.62). While STRAIGH (0.11) and AXSCO (0.14) have a low heritability.

**Table 2:** The broad-sense heritability (H^2^) values of architectural variables for the F1 population include the following: height of bole (BH), girth of stem (GIRTH), estimated bole volume (EBV), number of apical shoots (APNUM), number of terminal branches (BRCHTRM), tree straightness (STRAIGH), type of crown (TYCRO), axillary shoot score (AXSCO), axillary branch type (AXTYP), and diameter of axillary branches (AXDIAM).

Trait		H^2^
Quantitative	BH	0.58
	GIRTH	0.80
	EBV	0.84
	APNUM	0.60
	BRCHTRM	0.62
Qualitative	STRAIGH	0.11
	TYCRO	0.53
	AXSCO	0.14
	AXTYP	0.30
	AXDIAM	0.94

### Quantitative trait loci of architectural variables

**Fig 4:**
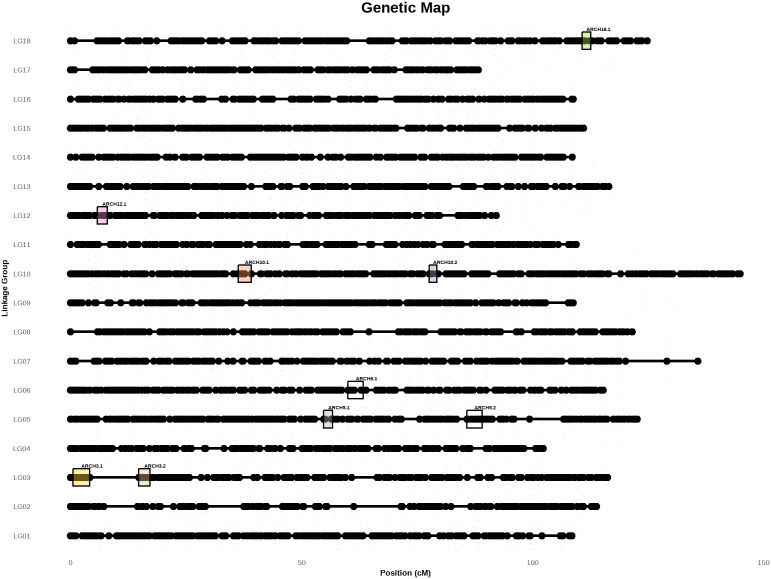
Position of chromosomal regions harbouring QTLs for architectural traits, on the high-density genetic map. ARCH3.1 represents the location of the QTLs of APNUM, EBV, TYCRO_CO and TYCRO_R traits, ARCH3.2 of EBV, GIRTH, AXDIAM_M, and AXDIAM_S and TYCRO_CO, ARCH5.1 of AXTYP_MO and AXTYP_MP, ARCH5.2 of AXTYP MO and AXTYP MP, ARCH6.1 of EBV and GIRTH, ARCH10.1 of AXTYP F, ARCH10.2 of EBV and GIRTH, ARCH12.1 of BRCHTRM, and ARCH18.1 of TYCRO R. See Table 3 for more information.

The QTL analysis was performed with the Kruskal-Wallis test for each quantitative variable and frequency of qualitative variable categories. Twenty quantitative trait loci were identified (S2 Table). Several of these QTLs colocalise in the same position revealing nine main chromosomal regions for four quantitative traits (APNUM, BRCHTRM, EBV, GIRTH) and six categories of three qualitative traits (TYCRO, AXDIAM, AXTYP) (Table 3). Two chromosomal regions were identified on LG3 (ARCH3.1 and ARCH3.2), LG5 (ARCH5.1 and ARCH5.2) and LG10 (ARCH10.1 and ARCH10.2) while only one chromosomal region was detected on LG6 (ARCH6.1), LG12 (ARCH12.1) and LG18 (ARCH18.1). QTLs for APNUM, EBV, TYCRO_CO, TYCRO_R traits colocalised on ARCH3.1 while ARCH3.2 harboured QTLs for EBV, GIRTH, AXDIAM_M, AXDIAM_S and TYCRO_CO traits. On LG5, QTLs for AXTYP_MO and AXTYP_MP traits colocalized on two positions ARCH5.1 and ARCH5.2. QTLs for EBV and GIRTH colocalized on ARCH6.1. On LG10, ARCH10.1 harboured a unique QTL for AXTYP_F and QTLs for EBV and GIRTH colocalized on ARCH10.2. On LG12 and LG18, ARCH12.1 and ARCH18.1 harboured a single QTL for BRCHTRM and TYCRO_R, respectively. The K values were the highest value for traits that colocalize in the same position of these QTLs. These K values vary from 12.33 to 20.375. These nine chromosomal regions were positioned on the high-density genetic map established by Ismawanto and collaborators [54] (Fig 4).

**Table 3:** Summary of chromosomal regions harbouring significant QTLs identified for tree architectural traits calculated with Kruskal-Wallis. The K value is the highest value for traits that colocalize in the same position. LG = linkage group; SNP = single nucleotide polymorphism; K = Kruskal-Wallis statistic K test.

Chromosomal region	Trait	LG	Start		Peak		End		K	p-value
			Marker	Position (cM)	Marker	Position (cM)	Marker	Position (cM)		
ARCH3.1	APNUM, EBV, TYCRO_CO-R	3	SNP0759	0.548	SNP0772	2.195	SNP0790	4.114	18.848	0.0001
ARCH3.2	EBV, GIRTH, AXDIAM_M-S, TYCRO_CO	3	SNP0791	14.728	SNP0792	15.277	SNP0816	17.197	15.57	0.0001
ARCH5.1	AXTYP_MO-MP	5	SNP1770	54.770	SNP1774	56.429	SNP1780	56.703	17.9	0.0001
ARCH5.2	AXTYP_MO-MP	5	SNP1924	85.772	SNP1930	87.695	SNP1939	89.067	17.545	0.0001
ARCH6.1	EBV, GIRTH	6	SNP2229	60.058	SNP2242	62.816	SNP2244	63.364	14.579	0.0005
ARCH10.1	AXTYP_F	10	SNP3812	36.332	g10A2316	37.705	SNP3820	39.090	20.375	0.0001
ARCH10.2	EBV, GIRTH	10	SNP3942	77.658	SNP3944	77.932	SNP3951	79.311	16.493	0.0001
ARCH12.1	BRCHTRM	12	SNP4585	5.772	SNP4596	7.42	SNP4598	7.968	14.821	0.0005
ARCH18.1	TYCRO_R	18	SNP7128	110.69	SNP7134	112.617	SNP7134	112.617	12.33	0.0005

### Analysis of chromosomal regions underlying QTLs

The nine chromosomal regions harbouring the QTLs were retrieved and annotated using the high-quality genome of clone PB 260 (Table 4). The size of these chromosomal regions ranged from 455,632 to 2,326,592 bp. These regions contain from 15 to 202 genes. ARCH3.1, ARCH3.2 and ARCH12.1 have the largest number of genes: 202, 170 and 81, respectively. Five associated regions having genes without significant functional enrichment (ARCH3.2, ARCH5.1, ARCH6.1, ARCH10.1 and ARCH10.2). Genes with significant GO term enrichment were found for four of the nine chromosomal regions (ARCH3.1, ARCH5.2, ARCH12.1, ARCH18.1). The frequency of GO terms associated with the genes within the chromosomal regions was calculated to identify over- and under-represented annotations in chromosomal regions (S3 Table).

**Table 4:** Physical position and GO term enrichment of genes underlying the nine chromosomal regions associated with QTLs detected for seven architectural traits.

Chromosomal region	Trait	LG	Physical position of chromosomal region (bp)			Total genes in the interval	Significant GO term enriched in chromosomal regions	Genes with significant GO term enrichment	Under-represented		Over-represented		Genes associated with development
			Start	End	Length	(N°)	(N°)	(N°)	GO (N°)	Gene (N°)	GO (N°)	Gene (N°)	(N°)
ARCH3.1	APNUM, EBV, TYCRO_CO, TYCRO_R	3	269,678	2,021,104	1,751,426	202	21	36	0	0	21	36	5
ARCH3.2	EBV, GIRTH, AXDIAM_M, AXDIAM_S, TYCRO_CO	3	2,354,001	4,680,593	2,326,592	170	0	0	0	0	0	0	0
ARCH5.1	AXTYP_MO, AXTYP_MP	5	16,327,902	17,433,681	1,105,779	33	0	0	0	0	0	0	0
ARCH5.2	AXTYP_MO, AXTYP_MP	5	97,634,289	99,144,017	1,509,728	52	4	15	0	0	4	15	5
ARCH6.1	EBV, GIRTH	6	77,267,404	78,308,329	1,040,925	15	0	0	0	0	0	0	0
ARCH10.1	AXTYP_F	10	60,132,594	61,281,788	1,149,194	27	0	0	0	0	0	0	0
ARCH10.2	EBV, GIRTH	10	71,663,821	72,119,453	455,632	36	0	0	0	0	0	0	0
ARCH12.1	BRCHTRM	12	2,830,734	3,521,994	691,260	81	25	26	0	0	25	26	5
ARCH18.1	TYCRO_R	18	29,846,327	31,489,780	1,643,453	64	10	24	2	12	8	12	4

Of the 680 genes found in chromosomal regions under QTLs (Table 4), 627 genes have a functional annotation (S3 Table). One hundred and one genes are associated with significant GO term enrichment (Table 4). Among these genes, 53 are directly or indirectly involved in plant development processes (S4 Table), including 19 genes playing a direct and major role in plant development which are carried by four chromosomal regions. The functions found for genes directly associated with growth and development processes were listed in Table 5. ARCH3.1 harbours five genes encoding xyloglucan fucosyltransferase, GDSL esterase/lipase GLIP1-5/GLL25, glycosyltransferase-like KOBITO 1, O-methyltransferase COMT-type and pectin esterase. All of these aforementioned genes are involved in cell wall development and modification, and in anther and pollen development. ARCH5.2 consist of five genes involved in growth and development through hormone signalling, cell morphology, trichome formation, flower and vascular development, secondary growth and stress responses (transcription factor BHLH95-like, MYB Transcription Factors, bHLH transcription factors HECATE 3 (HEC3), WRKY transcription factor 21 and WUSCHEL-related homeobox 4). ARCH12.1 harbours five genes encoding Three Amino acid Loop Extension (TALE) homeobox, Inositol-polyphosphate 2-kinase, transcription factor INDEHISCENT (IND/bHLH), FHY3/FAR1 family and Zinc-finger homeodomain protein 2. All the genes involved in plant growth and development like leaf morphology, shoot development, pollen and pollen tube development, chlorophyll biosynthesis and responses on stresses. Finally, ARCH18.1 harbours four genes involved in vegetative and reproductive growth such as shoot apical meristem development, signal integrator, plant morphology and response to environment (MAD box protein, Radical Shoot Apical Meristem (SAM) protein, basic helix-loop-helix leucine zipper transcription factor and transcription factor IND/bHLH).

**Table 5:** List of functions found for genes underlying chromosomal regions, which are associated with growth and development processes.

Chromosomal region	Position	Function	Characterised developmental function	Reference
ARCH3.1	PB260_LG3_002287	Xyloglucan fucosyltransferase	Plant cell wall biosynthesis	[63]
	PB260_LG3_000104	GDSL esterase/lipase GLIP1–5/GLL25	Anther and pollen development	[64]
	PB260_LG3_002334	Glycosyltransferase-like KOBITO 1	Cell elongation and cellulose synthesis	[65]
	PB260_LG3_000030	O-methyltransferase COMT-type	Involvement in lignin biosynthesis in pine xylem	[66]
	PB260_LG3_000096	Pectinesterase	Regulating cell wall modification and fruit ripening	[67]
ARCH5.2	PB260_LG5_000892	Transcription factor BHLH95-like	Negative regulator of trichome formation	[68]
	PB260_LG5_001411	MYB Transcription Factors	Growth and development, stress responses, hormone signalling, cell morphology, and pattern building	[69]
	PB260_LG5_000898	bHLH transcription factors HECATE 3 (HEC3)	Modulator of auxin and cytokinin responses to control development, flower development	[70]
	PB260_LG5_000882	WRKY transcription factor 21	Involve in abiotic response, developmental, and hormone-regulated processes	[71]
	PB260_LG5_000887	WUSCHEL-related homeobox 4	Regulate vascular cambium activity and secondary growth	[72]
ARCH12.1	PB260_LG12_000145	Three Amino acid Loop Extension (TALE) homeobox	KNOTTED1-like homeobox (KNOX) role in leaf, shoot apical meristem and seed development and respond to biotic and abiotic stresses	[73]
	PB260_LG12_000140	Inositol-polyphosphate 2-kinase	Pollen development, pollen tube guidance and embryogenesis	[74]
	PB260_LG12_001605	Transcription factor IND/bHLH	Key processes in plant growth and development in response to environment	[75]
	PB260_LG12_000161	FHY3/FAR1 family	Essential for phytochrome A controlled far-red responses chlorophyll biosynthesis	[76]
	PB260_LG12_001585	Zinc-finger homeodomain protein 2	Roles in plant growth, development, and stress responses	[77]
ARCH18.1	PB260_LG18_001654	MAD box protein	Essential transcription factor for vegetative and reproductive growth	[78]
	PB260_LG18_001127	Radical Shoot Apical Meristem (SAM) protein	Influence shoot apical meristem development	[79]
	PB260_LG18_001132	Basic helix-loop-helix leucine zipper transcription factor	Integrator of internal and external signals, controls the plant morphology	[80]
	PB260_LG18_001652	Transcription factor IND/bHLH	Key processes in plant growth and development in response to environment	[75]

## Discussion

The study of architectural traits is not trivial due to the evolution of these traits over time, the influence of the environment (competition between trees, soils, weather, etc.) and ageing as well as mutations that accumulate as trees grow [81–83]. For instance, trunk growth can be affected by the seasons, age of trees, and latex harvesting. Tree height also varies during the immature phase, but competition between trees influences their development, and after seven years of growth, heights were similar in the presented trial. Due to the cost of repeated observations, this observation date was therefore chosen because the trees had reached a certain level of maturity and their main characteristics (number of apical branches, canopy, etc.) were well established. Of course, the results presented will need to be validated by large-scale clonal trials, but genetic analysis of a population can only be carried out within the framework of small-scale clonal trials.

This genetic analysis of quantitative and qualitative architectural traits of rubber trees in a segregating population revealed the high heritability of seven traits, making it possible to develop breeding strategies for agronomic and environmental factors. These factors include the role of the crown, particularly in agroforestry systems, in promoting light for intercropping and improving the microclimate under trees, tolerance to wind damage, which is a major factor in the adaptability of trees to climate change, and the influence of tree architecture on wood and latex production. This study is also the first to attempt to identify the genes underlying the QTLs and to gain a better understanding of the developmental functions involved.

### Seven of the ten architectural traits showed high heritability, revealing potential applications in breeding

The high heterozygosity and allogamy of *Hevea brasiliensis* have often led to high variability in F1 genetic populations for agronomic traits such as drought tolerance [84], leaf disease resistance [85] and latex production [54], as well as the susceptibility to Tapping Panel Dryness [86]. The distribution of architectural traits showed the same high variability in the F1 population and parent clones, previously observed by Korieocha and collaborators for agro-morphological traits of 16 rubber genotypes [87].

The large segregating population used in this study made it possible to accurately measure heritability. Seven of the ten architectural traits were identified with high broad-sense heritability (H^2^ > 0.5), highlighting the significant genetic contribution to their variation. Quantitative traits such as trunk girth, estimated bole volume, and in particular the diameter of axillary branches have a very high heritability (>0.80). These traits are particularly important for breeding programmes and were previously associated with yield [88–91]. Bole height, the number of apical branches, the abundance of terminal branches, and the crown type had a more stable phenotype and intermediate heritability (0.58, 0.60, 0.62 and 0.53, respectively). Some qualitative traits do not have stable phenotypes among genotypes and parent clones. Tree straightness, the abundance of axillary branches and the orientation of axillary branches gave a particularly inconsistent phenotype among the 10 trees observed per genotype, and had low heritability (0.11, 0.14 and 0.30, respectively), indicating a greater influence of the environment on phenotypic variation.

The type of crown is also an essential factor associated with trunk girth and branching. The PCA analysis showed three main correlation groups among these traits. First, genotypes with round-type crowns (TYCRO_R) have higher girth, thicker branches (AXDIAM_M), and more apical and terminal branches. Gireesh and Mydin showed that trees with large girth often form a globose or round canopy [7]. Secondly, broom-type crown (TYCRO_B) was correlated in this study with orthotropic branching. These results are supported by other authors who have also observed a correlation between broom-type crown and orthotropic branches with narrow angles [9], and multiple apical shoots resembling fan-shaped [92]. Finally, genotypes with conical crowns (TYCRO_CO) generally have thinner branches (AXDIAM_S) and vertical oriented branches (AXTYP_MP) with wider angles [44]. This corresponds to cone-shaped described by Clément-Demange and collaborators [92].

The three crown types characterised above have a good heritability, making this trait suitable for breeding, particularly for rubber-based agroforestry systems and tolerance to wind damage. Phenotypic variations in girth are also laying the groundwork for the development of fast-growing clones for early tapping, clones with high-biomass (timber clones) and latex-timber clones that support both wood and latex production.

### Selection of the type of crown for specific intercropping systems

Tree canopy architecture plays an important role in intercropping systems such as agroforestry by regulating light penetration into the understorey, which affects temperature and humidity, ultimately creating a cooler microclimate for better habitat quality and the growth of intercropped crops [5,6,93]. Although many studies have reported that light in rubber agroforestry systems is reduced as canopies grow, little is known about specific rubber crown types and light availability in intercropping. The design of the small-scale clone trials in this study did not make it possible to analyse the impact of shading of the three crown types on light penetration. Indeed, interactions between trees promote complementarity between crowns and can thus modify the structure of the canopy [94]. This will require large-scale clone trials (1 ha per clone) to be set up in order to analyse the conditions under the trees (microclimate and light). This type of trial is being prepared as part of a participatory breeding project [27]. Several clones with contrasting canopies could be tested. Of the 189 genotypes, 26 have a round-type crown (TYCRO_R), 47 a broom-type (TYCRO_B), and 64 a conical-type (TYCRO_CO). Although heritability is high (0.53), the other genotypes exhibit an inconsistent phenotype revealing the influence of environment including the rootstock, as has been shown in fruit trees such as apple trees [95].

Work on other species clearly demonstrates the value of certain canopy structures for agroforestry systems. A study on shaded cocoa systems offers insight into various canopy types that can influence light access in agroforestry [4]. These authors identified several crown types in cocoa agroforestry systems: ranging from cylindrical, elongated to rounded, upswept (vase-shaped), conical to pyramidal, and spade-shaped. They use crown diameter, volume and area as essential variables for classifying these types, which directly affect shade and light availability for understorey growth in agroforestry. Asante and collaborators divided crown types into three shading potential clusters: high, moderate and low, each affecting light availability and understorey growth [4]. High shading-potential crowns, ranging from cylindrical and elongated to rounded, corresponding to TYCRO_R in this study, have large diameters and volumes that provide dense shade that is ideal for shade-sensitive crops by stabilising microclimate conditions. Moderate shading-potential crowns such as upswept or vase-shaped, corresponding to TYCRO_B, offer balanced shading with moderate diameter and volume, supporting a mix of sun and shade-tolerant crops. Low shading-potential crowns, including conical to pyramidal and spade-shaped types (TYCRO_CO), feature smaller diameters and volumes that allow more sunlight penetration, suitable for sun-loving crops such as corn, rice chilli, etc., by reducing light competition in the understory.

In addition to breeding, changing rubber planting systems from a standard single-row system to a double row system with wide spacing between the double rows enhances light availability for understorey crops [96]. The double-row system makes it possible to have more than 80% of light penetration during the growth period of mature plantations. Huang and collaborators similarly found that the double-row system, while slightly reducing rubber yield per hectare, maintained yield per tree and provided two to four hours of direct sunlight in intercropped areas, fostering growth of sun-loving crops [97]. However, trees of mature plantations of clone GT1, even with wider inter-row spacing in the double-row system, expand their canopies and create significant shade, which reduces yields of shade-sensitive intercrops like lemon and cola after nine years of plantation [36]. In contrast, clones with compact, upright growth habits, such as CATAS 7-20-59, maintain limited canopy spread in wider spaces, allowing better light conditions for intercropped plants [97]. These growth habits and crown types are essential for selecting rubber clones suitable for long-term intercropping systems, particularly in the double-row system. Conducting large-scale clone trials with contrasting crown types should help to better understand the influence of the crown types.

### Architecture characteristics prone to wind tolerance

Rubber tree clones exhibit distinct wind resistance based on crown architecture. An example of a wind-resistant clone is GT1. Trees from clone GT1 have a compact, brush-shaped crown and slow vertical growth. In contrast, clones PB 235 and RRIM 600 are wind-susceptible. Clone PB 235 has a cone-shaped crown, initially remains stable but becomes wind-susceptible as its heavy crown creates balance issues over time. This change occurring during the growth of mature trees indicates that breeders must take into account the evolution of architectural traits over a long period of growth. Clone RRIM 600 forms long forking branches and dense upper structures, which lead to imbalance because of the top-heavy crown [44,92]. Further studies revealed that the structural characteristics of clone GT1 displayed a twice higher safety factor to wind firmness than PB 235 [98]. The larger crown of PB 235 generates a drag force 70% greater than GT1 that explains the wind susceptibility of clone PB 235. This is in line with advanced studies using LiDAR (LIght Detection And Ranging) and TLS (Terrestrial Laser Scanning) technologies [9,99]. According to these studies, clone CATAS 7-20-59 is classified as the most tolerant clone. This clone shows a compact vase-shaped crown with narrower branch angles and stable wind flow that is more resilient to wind damage. This phenotype could correspond to the phenotype AXTYP_MO for orthotropic branches in this study, a phenotype that is associated with TYCRO_B for the broom-shaped crown. In contrast, PR 107 was identified as wind-susceptible due to its large spread-out crown, wider branch angle, and high leaf area index, which result in greater wind loads. In India, the wind-susceptible clone RRII 105 has a large and globose canopy [7]. Plantations with the latter clone show a high loss of trees from wind damage over 18 years.

In this study, tree height was not measured because there were not obvious differences between genotypes in the F1 population when the trees reached a certain maturity with canopy closure after two years of tapping. Unlike most studies observing tree growth and height in high-density seedling evaluation trials, we have carried out phenotyping of architectural traits in a small-scale clone trial with normal density, promoting growth comparable to that of commercial plantations. However, the crown types were contrasted at this stage of tree growth. The TYCRO_CO is similar to the description of clone PB 235 [44,92] and PR 107 [9,97]. These wind-susceptible clones are initially tall with a single-trunk structure. During the mature period, height and branch mass increase to confer their wind susceptibility. The TYCRO_R is similar to clone RRII 105 [7]. This clone shows large crown size with mixed branch orientation, and tends to span neighbouring rows, leading to its high susceptibility to wind damage. The classification of TYCRO_B remains uncertain due to the lack of measurements for branch and crown dimensions. It could potentially align with either the wind-susceptible fan-shaped crown of clone RRIM 600 [92] or the wind-tolerant vase-shaped crown of clone CATAS 7-20-59 [9]. This observation does not make it easy to establish a choice of architectural structures that could be selected to promote tolerance to wind damage.

For double-row systems, the open areas of the wide spacing may also affect wind resistance because wind speed and turbulence can be greater in these spaces. As described by Cheng and collaborators, wind tunnel observations show that wider gaps between rows increase the risk of wind damage by reducing wake overlap, accelerating wind speed recovery, amplifying turbulence and diminishing aerodynamic drag, all of which weaken the effectiveness of tree stands in reducing wind speed, thereby increasing the potential for damage caused by strong winds [100].

Breeding for tolerance to wind-damage is not yet obvious. It requires the control of the size and form of crown, and the height and width of the trees in relation to the cropping systems, balance between the root-scion parts of the tree and root anchoring. Most of the above studies were done for standard density and monoculture cropping system either in large-scale clone trials or commercial plantations. In addition, rubber scion clones are grafted on non-selected seedling rootstocks, which all have different genotypes. Rootstocks are known to control the vigour of scions in fruit tree species [101]. Consequently, it is difficult to control the balance between the root system and the aerial part. Despite these constraints, some rubber clones have shown reliable and consistent architectural characteristics regardless of the location and cultivation conditions. Further efforts are necessary to characterise and model the best combination of architectural traits for wind tolerance.

### Architecture traits for biomass and latex yield

The trunk girth and the volume and the height of the bole are essential variables for assessing biomass and latex yield in rubber trees. Girth has the most direct impact on latex yield, making it a key focus in breeding programmes, along with bark thickness and latex vessel rings [89]. Girth also demonstrates high heritability and a positive yield correlation, supporting the selection for larger girth to enhance latex production [90,91]. The production cycle of rubber plantations is around 25 years. They produce substantial post-harvest biomass, including fuelwood and timber for plywood and other products, thus supporting both energy and timber markets [102,103]. In this study, GIRTH and EBV showed a high broad-sense heritability (> 0.80), indicating strong genetic control. Larger girth is correlated with greater bole volume and thicker axillary branches, and 61 genotypes scored high on these traits (see Fig 2), making them strong candidates for developing latex timber clones that combine high latex yield with valuable timber properties for agroforestry [104].

Selective breeding in rubber trees can enhance traits essential for latex and timber production [105,106]. Gouvêa and collaborators demonstrated that selecting young trees for high latex yield and trunk girth enables early identification of trees with strong potential for profitable dual use, benefiting both rubber and wood production when trees are mature [105]. Chaendaekattu and Mydin further noted that selecting for girth not only enhances wood traits like fibre diameter and wall thickness but also maintains wood density, though it may reduce fibre length [106]. Thus, focusing on both girth and latex yield can optimise rubber trees for dual-purpose production, balancing latex yield with wood quality.

### Towards a better understanding of the regulation of architectural traits in rubber trees

Numerous studies on the fundamental components of branch architecture as well as genetic and hormonal influences are being conducted on model plant systems and trees with non-standard architectures [107]. Recent discoveries on the regulation of shoot architectures have made it possible to identify the genes for dwarf, drooping, columnar, and pillar growth habits [108]. The genetic pathways that determine tree architecture in different species, with transcription factor genes that regulate growth cycles, DELLA genes that modulate stature [107], and TILLER ANGLE CONTROL 1 (TAC1) [109] and LAZY1 genes that control branch angles and orientation [107]. Although few studies have been conducted on tropical trees, some studies on rubber trees have made it possible to characterise histone acetyltransferases (HbHATs) and histone deacetylases (HbHDACs) in tree development [110], *Flower locus T-like* genes such as HbFT1 in flowering [111], and the molecular interplay between jasmonic acid and brassinosteroid on vascular development and its impact on laticifers [112]. It is interesting to note that several functional analyses carried out on transgenic rubber trees have shown that the overexpression of genes involved in ROS-scavenging systems and an Ethylene Response Factor, acting at the crosstalk between the ethylene and jasmonate signalling pathways, induces faster growth of the aerial parts and roots than in wild plants [113–115].

To our knowledge, this genetic analysis is the first study to cover all the main architectural traits of rubber trees and to identify candidate genes involved in the development of this species. Most genetic studies have focused on growth and production traits, as well as tree height at an early stage of growth [46–50,106]. Several studies using high-density genetic maps have even identified genes underlying QTLs for growth traits [45,51,116].

Of the nine chromosomal regions covering QTLs detected in this study, four harbour genes with significant GO term enrichment. For ARCH3.1, the QTLs for the APNUM, EBV and TYCRO_CO/R traits colocalise on this chromosomal region and are intercorrelated according to the PCA. Thus, the number of apical branches (APNUM) is positively correlated with the volume of the main trunk (EBV) and TYCRO_R, and negatively correlated with TYCRO_CO. Analysis of the function of the five genes present in the ARCH3.1 chromosomal region revealed relevant roles related to these traits. Xyloglucan fucosyltransferase is involved in cell wall biosynthesis and mutants with impaired xyloglucan biosynthesis often exhibit dwarfism, reduced branching, or altered growth patterns [117]. Similarly, GDSL lipases are upregulated under biotic/abiotic stress, thereby influencing how trees prioritise between vegetative and reproductive growth. Another gene encoding glycosyltransferase KOBITO 1 participates in cell elongation and cellulose synthesis. KOBITO 1 mutants in Arabidopsis exhibit reduced cell expansion and dwarfism, illustrating the importance of this gene in vertical and radial growth [118]. It is interesting to note that this gene has also been identified under a growth QTL in rubber trees [45]. COMT-type O-methyltransferase is involved in lignin biosynthesis. The suppression of COMT leads to a change in lignin content, which has an impact on wood strength and stem morphology [119]. Finally, genes encoding pectinesterase are involved in regulating cell wall modification and fruit ripening, and are often co-expressed in zones of active cell growth, including developing stems and branches [120]. ARCH5.2 harbours genes of a unique QTL associated with AXTYP_MO/MP for orthotropic and plagiotropic axillary branches. Orthotropic branches are a characteristic of juvenile rubber trees [121]. Several gene functions found in this study are directly involved in orthotropy via vascular development and possibly connected to juvenility via cambium age-related changes. WUSCHEL-related homeobox 4 (WOX 4) regulates vascular cambium activity and secondary growth [72]. Thus, WOX4 could contribute to phase changes in stem/branch growth. In the same way, WRKY21 is functionally relevant in maintaining juvenility and possibly influencing vertical vs lateral growth via hormonal balance [71]. A member of the bHLH family, HECATE 3, is a key regulator of flowering transition through cytokinin-auxin balance, which is also crucial for juvenile-to-adult phase change. ARCH12.1 is also a chromosomal region associated with a single QTL for BRCHTRM. The number of terminal branches in a tree reflects branching complexity and shoot meristem activity. Of the five identified genes involved in plant development, KNOX is a critical regulator of meristem maintenance, shoot architecture and branching pattern through its role in shoot apical meristem [122]. ARCH18.1 harbours genes underlying a single QTL for TYCRO_R. This chromosomal region comprises four genes with significant GO terms involved in developmental processes and four directly linked to this phenotype of round crown type: a transcription factor for vegetative and reproductive growth (MAD box protein), a Radical Shoot Apical Meristem (SAM) protein, a Basic helix-loop-helix leucine zipper transcription factor, and an IND/bHLH transcription factor. SAM protein is an integrator of internal and external signals for plant morphology and key process in plant growth and development and response to environment [123].

Five of the nine chromosomal regions did not show any significant GO term enrichment. However, they harbour numerous genes that should be carefully in-silico characterized. The genetic approach used in this study enabled us to identify genes that would have been more difficult to identify using another approach, as we observed a mixture of orthotropic and plagiotropic branches. The identification of these candidate genes raises the question of their functional validation and the use of this knowledge in breeding programmes. Functional analysis of candidate genes is possible in transgenic rubber trees thanks to an effective *Agrobacterium tumefaciens*-mediated genetic modification method [124,125]. However, confinement in greenhouses does not allow the characterisation of mature trees for their tree architecture. To date, only a few field trials of transgenic rubber trees have been established in India and Malaysia [126]. The stability of the QTLs identified in this study could also be investigated in new biparental or GWAS (Genome-Wide Association Studies) populations.

## Conclusions

This study is the first genetic analysis of architectural traits in rubber. Among the studied traits, trunk girth, estimated bole volume, and eight categories of qualitative traits linked to axillary branch diameter, branch type and crown type have a high heritability. Literature evidence suggests that these traits may be used to select genotypes with contrasting architectural types that will be used in a large-scale clone trial involving various cropping systems, including agroforestry and, in particular, double-row systems to study their impact on light penetration and microclimate under trees. The annotation of chromosomal regions made it possible to identify 19 candidate genes associated with developmental processes. This result confirms the accuracy of this genetic analysis, which was carried out using a high-density map and a new genomic sequence of clone PB260, the male parent of the F1 population. Our findings highlight the essential role of rubber tree architecture in better responding to the challenges associated with rubber cultivation and climate change.

## Supporting information

S1 Table LS-means of quantitative variables and frequency of qualitative data categories. (XLSX)

S2 Table Results of the QTL analysis performed with the Kruskal-Wallis test. Positions are in cM. K values and significance of each architectural variable and category. Values highlighted in pink are k values greater than 10, and in yellow are the regions chosen as QTL.
(XLSX)

S3 Table List of genes with gene ontology (GO) terms in QTLs. (XLSX)

S4 Table List of genes directly or indirectly associated with developmental processes for the various QTLs. (XLSX)
